# Comparative inhibitory effects of zeolite, sepiolite, and kaolinite on A549 lung cancer cells

**DOI:** 10.1371/journal.pone.0340270

**Published:** 2026-02-11

**Authors:** Fariba Nemati Shamsabad, Mohammad Hassan Salehi, Jalaleddin Shams, Tooba Ghazanfari

**Affiliations:** 1 Soil Science Department, College of Agriculture, Shahrekord University, Shahrekord, Iran; 2 Department of Oncology and Hematology, Shahed University, Tehran, Iran; 3 Immunoregultion Research Center, Shahed University, Tehran, Iran; Monash University, AUSTRALIA

## Abstract

**Background:**

Clays, by their unique structure, due to reduction in the acidity of blood and the surrounding environment, crystalline structure, pores, channels, and variations in microporosity significantly influence cellular growth. This study intends to examine the inhibitory effect of zeolite, sepiolite, and kaolinite on the human lung adenocarcinoma cell line (A549) in comparison to PBMCs (peripheral blood mononuclear cells) as representative cells of the immune system.

**Methods:**

The cytotoxic effect of zeolite, sepiolite, and kaolinite was assessed using cell viability assay, comparing responses in cancerous A549 cells and non-cancerous peripheral blood mononuclear cells (PBMCs) to determine selective toxicity.

**Results:**

Our results demonstrate that all three clays significantly decreased A549 cell viability in a dose- and time-dependent manner, while exhibiting minimal cytotoxicity toward PBMCs. The IC50 values were calculated as follows: 0.34 mg/mL for zeolite, 0.06 mg/mL for kaolinite, and 0.09 mg/mL for sepiolite.

**Conclusion:**

These findings suggest a degree of selective anticancer activity, positioning these clay minerals as promising candidates for further development as adjunct or alternative anticancer agents.

## 1. Introduction

Cancer is a major global health problem and the primary cause of death worldwide. Malignant tumors severely impact human health, and medication resistance is a significant factor contributing to the recurrence of the disease [1,2]. Chemotherapy continues to be the primary treatment method for various forms of cancer. Nevertheless, cancer cells acquire resistance to chemotherapeutic treatments and become unresponsive to their effects [3]. The presence of multidrug resistance in cancer cells requires investigating alternative and complementary therapeutic approaches [4,5]. Due to the introduction of new anticancer medications, the rate of cancer recovery has somewhat increased recently [6]. Researchers in the field of cancer are continuously working on the development of new and more effective surgical techniques, radiotherapy, and chemotherapy agents [7]. In this context, clays are emerging as systems with exceptional potential in cancer treatment. They are not only utilized as carriers for the delivery of various anti-cancer agents but have also been demonstrated to act as a natural anti-tumor active component in certain types of clays [8]. The type of clay can play a role in cell survival. Depending on the type, clays can create the highest porosity and correlation between mechanical properties and structural outcomes, contributing to the highest rate of cell inhibition [9].

One of the most widespread types of cancer is lung cancer, responsible for around 9.6 million deaths in 2018 [10] and accounting for the highest percentage (18.0%) of all deaths caused by cancer [11]. Although there have been advancements in diagnosing and treating lung cancer, therapy for this disease still poses challenges. The primary cause of therapy’s declining efficacy is the development of resistance to medication treatment [12], leading to a 5-year survival rate of ≤ 18% [13].

Clay minerals are widely employed to treat pain, open skin wounds, skin disorders, and gastrointestinal issues [14]. They are also employed as an adjuvant in anticancer treatment [15].

Researchers in the field of cancer are continuously developing novel surgical techniques, more effective radiotherapy, and chemotherapy drugs. Biological therapies, including monoclonal antibodies, cancer vaccines, and gene therapies, are also active areas of research. Additionally, anti-angiogenic drugs exist that can inhibit the growth of blood vessels necessary for tumors [7]. In this context, clays have gained attention as systems with remarkable potential in cancer treatment. These materials are not only being investigated as carriers for delivering various anti-cancer agents, but it has also been demonstrated that certain types of clays can act as natural components with anti-tumor activity [8].

The enhancement of the effects of the drug Prazquantel in interaction with montmorillonite and sepiolite resulted in a significant increase in the control of dissolution rates and the amount of soluble Prazquantel, thereby improving the bioavailability of this drug [16]. The interaction between curcumin, chlorpromazine (CPZ), and the antidiabetic drug metformin hydrochloride facilitated prolonged release and reduced side effects when incorporated within montmorillonite clay layers [17–19]. Additionally, montmorillonite clay was modified with a chitosan-polyethylene glycol blend, resulting in a biodegradable composite suitable for oral insulin delivery [20]. Kaolinite has demonstrated a soothing effect on inflammation [21], has been effective in treating bacterial wound infections [22], and can serve as a standard drug carrier, providing an advanced means for controlling drug release and ensuring the safety of oxaliplatin (OL), dexamethasone, antibiotics, 5-FU, doxorubicin, diclofenac, tetracycline (TC), and doxycycline (DC) [22–26]. Beta zeolite has been evaluated as an effective carrier for the drug mitoxantrone, enhancing cytotoxic activity against cancer cells [27].

The structure and composition of a particular clay mineral determine its physical and chemical qualities. The primary commercial clays, including kaolin (1:1 phyllosilicates), zeolites (tectosilicates), and sepiolite (2:1 inverted ribbons), have distinct structures and compositions. Octahedral and tetrahedral sheets are fundamental components of clay minerals. The arrangement and composition of these octahedral and tetrahedral sheets lead to variations in the clay’s physical and chemical properties [28].

Clay and clay minerals are different. The term “clay” refers to materials with a diameter ≤2 μm in equivalent spherical diameter [29,30], while according to the Joint Report Of The Nomenclature Committee, clay minerals refer to phyllosilicates [30]. Clay minerals are not only characterized by their high adsorption capacity, cation exchange capacity, colloidal capacity, surface reactivity, extension in water, and suitable rheological manners [31], but also by their diverse reactivity [32] and variations in viscosity, morphology, surface area, and degree of adversity [33]. This complexity makes them a fascinating subject of study.

Three factors determine the selection of a given clay for specific uses: its mineralogical composition, which refers to the kind of mineral it contains; the arrangement, which can be either a 1:1, 2:1, or 2:1:1 layer class; and the clay’s chemical content [34]. The predominant clay minerals utilized in medicinal compositions comprise bentonite, kaolinite, zeolite, and sepiolite.

A significant member of the kaolin group is kaolinite. Kaolinite is a 1:1 phyllosilicate with the chemical formula Al₂Si₂O₅ (OH)₄, or in oxide notation: Al₂O₃·2SiO₂·2H₂O. Kaolinite is dioctahedral, containing Al³⁺ in octahedral sites and Si⁴⁺ in tetrahedral sites. Typically, it is observed in layers of pseudohexagonal platelets less than two μm in size [35]. However, the size of the particles might range from 0.3 to 100 μm [36]. This clay mineral exhibits insolubility in water and acidic or alkaline hydroxide solutions [35]. Moreover, it exhibits a broad pH range [37], possesses strong lubricating properties [35], and is non-abrasive [37]. This non-swelling clay mineral is advantageous for several therapeutic applications in comparison to other clay minerals. In addition, it can endure temperatures as high as 400°C [38]. Kaolinite is a stable mineral even in acidic environments and has little moisture at equilibrium [39].

Sepiolites, with the chemical formula (Si₁₂O₃₀Mg₈ (OH)₄ (H₂O)₄ × 8H₂O), represent another group of phyllosilicates. Although classified as a 2:1 type, they possess a unique structure that serves as an intermediary between 1:1 and 2:1 minerals. Sepiolite is composed of 2:1 phyllosilicate ribbons that run parallel to the fiber’s c-axis. These ribbons exhibit intermittent inversions of apical oxygen in the tetrahedral sheet. These inversions occur at regular intervals of six silicon atoms. The porosity of the structure indicated above arises from two factors, including the intervals between the fibers or bundles, which have a PD greater than 15 A˚, in addition to the repetitive inversions of the silicate layer, which have a pore-effective diameter (PD) less than 15 A˚. Tunnels are acknowledged as a significant factor in determining the movement of solutes into and out of a system [40]. Sepiolite possesses a significant quantity of adsorption sites [41], as well as spacious tunnels, which facilitate higher rates of diffusion [42,43].

The general chemical formula of natural zeolite (clinoptilolite) is (Na,K)₆Al₆Si₃₀O₇₂·20H₂O, with a Si/Al ratio that can vary from 4 to 3.5 [44]. Zeolites are commonly used due to their porous nature, which means they have a substantial interior surface area of approximately 102 square meters per gram. Additionally, they possess ion-exchange capabilities. The adsorption characteristics of zeolites are determined by the capacity of adsorbate molecules to enter the spaces inside zeolite structures, which are restricted by their size and the dimensions of zeolite pores (varying from 0.4 nm to 1.3 nm). Adsorption can occur on both surfaces of zeolite, including the internal and external. Another intriguing scenario occurs when molecules of similar diameters to zeolite pore sizes are adsorbed. Pure silica zeolites do not exhibit ion-exchange properties. These materials can adsorb molecules that are neither acidic nor basic [45].

An analysis and examination of the impact of clay and clay minerals on viability would be valuable for forthcoming research on the potential usefulness of these minerals as adjuvants in anticancer therapy. Clays are increasingly used as a carrier of cancer drugs. Despite the studies conducted on the application of clays in cellular growth, the mechanisms of action of clays and the impact of their physical, chemical, and morphological properties on cellular growth have not received adequate attention. Therefore, the present research aims to investigate the cellular response to these particles, with a particular emphasis on the type and size of clay particles and their action.

Zeolite, sepiolite, and kaolinite exhibit diverse morphologies, including plate-like, tubular, spherical, and fibrous forms, and possess tunable physicochemical and morphological properties that make them exceptional candidates for delivering therapeutic agents to tumor sites. Notably, these layered minerals serve as effective nanocarriers with drug loading capacities typically ranging from 1% to 10% by weight, providing enhanced stability and controlled release of anticancer agents. Their biocompatibility and structural versatility underlie their growing use in cancer research and therapy. Therefore, the current research aimed to scrutinize the inhibitory potential of these clays on the viability of A549 cells (a human lung adenocarcinoma cell line) in comparison with the PBMCs as representative immune cells. An in-depth look at how clay and clay minerals affect normal and cancerous cell viability would be very advantageous for future research on the possible application of clay minerals as adjuvants in anticancer therapy.

## 2. Materials and methods

### 2.1. Clay minerals

Kaolinite, sepiolite, and zeolite were obtained from natural mines in Iran (kaolinit, natural mines in Hamadan; sepiolite, natural mines in Fariman; and zeolite, natural mines in Semnan, Iran). We acquired the natural kaolinite and zeolite in 325 and 250 mesh sizes from ZAMINKAV Engineering Company, Iran. The sepiolite was extracted from the Fariman mine with a particle size of 270 meshes.

The purity of clays was evaluated by examining the X-ray diffraction patterns of the minerals using the Bruker D8-Advance XRD instrument. The clay compositions were analyzed, and the presence of toxic metals was detected using X-ray fluorescence (XRF). The determination of elements using XRF is primarily aimed at demonstrating the minimal presence or absence of heavy metals. This finding indicates the non-toxicity and safety of the clay.

The sterilization of clays was performed by means of a γ-ray using the γ-Cell 220 instrument. To achieve this objective, the samples underwent laboratory culturing. The elimination of the identified bacterial and other contaminants was done using a 30-kG dose of γ-rays.

The zeolite CEC (cation exchange capacity) varies because of its unique structure, including the external and internal CEC. In summary, a solution of sodium acetate (30 mL) with a concentration of 1 M and a pH of 5 was combined with zeolite (2 g). The resultant mixture was subsequently rinsed with water and alcohol four times. Subsequently, the tetra-butyl ammonium chloride (30 mL, 0.5 M) was added, and then the samples were subjected to 60 °C for 24 hours, and finally, the supernatant was gathered in a flask. A flame photometer was used to measure the sodium content to determine the non-zeolite CEC. Next, the excess tetra-butyl ammonium chloride was removed using washing with 95% alcohol and subsequently with a solution of 1 M ammonium acetate three times. Ultimately, the liquid portion was gathered to ascertain the sodium concentration and compute the zeolite’s internal CEC [46]. The CEC of kaolinite and sepiolite and the external CEC of zeolite were assessed using ammonium acetate at a pH of 8.2 [47].

For use in cell culture, suspensions were prepared by dispersing the powders in sterile phosphate-buffered saline (PBS) at specified concentrations under sterile conditions. Suspensions were subsequently sterilized by filtration through a 0.22 µm filter prior to cell treatment. Cells were treated with clay suspensions for 48 hours at ranging concentrations.

### 2.2. A549 cell line and PBMC preparation

This study was conducted following ethical standards approved by the Shahed University Ethics Committee under the code IR.SHAHED.REC.1401.161.

#### 2.2.1. A549 Cell culture.

The A549 cell line, derived from the epithelial lung cancer tissue of a 58-year-old Caucasian male, was isolated in 1972 through the culture of tumor tissue samples and subsequently donated to the ATCC (American Type Culture Collection) [48]. These cells are characterized by their squamous nature and exhibit spindle-shaped morphology. Under laboratory conditions, they grow as a monolayer and exhibit adhesive properties. The A549 cell line was obtained from the Pasteur Institute Cell Bank in Tehran, Iran. The A549 cells were cultivated in RPMI 1640 media, enriched with 10% FBS and 50 U/ml penicillin, and incubated at 37 °C in an environment containing 5% CO_2_.

#### 2.2.2. PBMCs isolation.

The PBMC isolation was done using density gradient centrifugation with Ficoll-Hypaque. Specifically, the PBS solution was mixed with heparin coagulant in a 1:1 ratio. Next, the blood sample was carefully poured on top of the Ficoll-Hypaque (Sigma, St. Louis, MO, USA). Lastly, PBMCs were isolated from the fluid by employing density centrifugation at 400 g for 25 minutes. The viability of the PBMCs was determined using trypan blue staining.

### 2.3. Treatments

Finding the right metrics to evaluate and contrast therapy efficacy in cancer research is crucial. At this time, determining the half-maximal inhibitory concentration (IC50) has become more important. A metric used to evaluate the efficacy of possible medicinal drugs is the IC50 value, which represents the concentration of a substance at which 50% of cell viability is suppressed. Because it provides a quantitative measure in cell viability experiments, researchers may compare the effectiveness of various substances and make well-informed decisions when developing cancer treatments [49].

The inhibitory effect of clay minerals on the viability of the A549 cell line was assessed as follows: The cells were distributed at densities of 20,000 and 200,000 cells per well for the A549 cell line and PBMCs, respectively. To determine effective concentrations, the half-maximal inhibitory concentration (IC50) was calculated. In IC50 assessments, the highest concentration typically tested is one milligram per milliliter. Dilutions are performed in a one-to-two manner; based on the IC50 values and the reviewed literature, the concentrations for the study were selected. They were then exposed to various concentrations of clay (0.5, 0.1, 0.05, 0.01, 0.005, 0.001, 0.0005, and 0.0001 mg/mL) for 48 hours in a 5% CO_2_ incubator at 37 °C after passage 2. In *in vitro* studies, various durations, such as 24, 48, or 72 hours, are utilized for testing purposes. In our pilot study, a 48-hour duration exhibited greater stability (less variance), and the results from the wells were more closely aligned. Therefore, a 48-hour timeframe was selected for these studies. The control sample consisted of untreated cells. All the experiments were replicated three times. The A549 and PBMC cell viability was assessed using the MTT method [50].

### 2.4. MTT assay

The MTT assay is an indirect method for evaluating cell viability and proliferation, as the absorbance at a wavelength of 570 nm can be correlated with cell number. MTT is a colorimetric method based on the reduction and cleavage of yellow MTT (3-(4,5-dimethylthiazol-2-yl)-2,5-diphenyltetrazolium bromide) crystals by the enzyme succinate dehydrogenase in living cells, resulting in the formation of insoluble purple formazan crystals. The color of the tetrazolium compound MTT is manifested in viable cells. Mitochondrial dehydrogenases in living cells cleave the tetrazolium ring, producing NADH and NADPH, which leads to the formation of a purple precipitate known as formazan. This precipitate can be dissolved using acidic isopropanol. Conversely, dead cells lack this capability and cannot perform this function. In this assay, the purple color indicates the presence of viable cells, and the intensity of the color correlates with cell number.

The procedure for conducting the MTT assay after 48 hours of cell incubation is as follows:

Initially, 5 grams of yellow MTT powder were dissolved in one milliliter of PBS (a concentration of 5 mg/mL is required). Next, the stock solution of MTT at a concentration of 10 µL was added to 100 µL of culture medium in all wells (20 µL per well), and the culture plates were incubated again at 37 degrees Celsius for 4 hours. After the incubation period, the medium containing MTT was removed, and to dissolve the formazan crystals, 100 µL of acidic isopropanol (0.04 M HCl in isopropanol) was added to each well, followed by pipetting. Absorbance at 570 nm and reference absorbance at 630 nm were measured using an ELISA reader. The data were presented as the rate of inhibition of cell proliferation, evaluated according to the following formula:


$$\text{Cytotoxicity}\;(\%)=\text{100}-\frac{\text{Cell}\;\text{OD}\;}{\text{Control}\;\text{OD}}\times \text{1}\text{0}\text{0}$$

where:

Cytotoxicity = Rate of inhibition of cellular proliferation

Cell OD = value of cells with Clay treatment

Control OD = value of untreated cells

### 2.5. Statistical analyses

The statistical analysis was performed using GraphPad Prism software, version 9. The data are presented as the mean ± SEM (standard error of the mean) of the three replications. The normality of the data was assessed using Anderson-Darling tests. The one-way ANOVA test was employed to compare cell viability groups after treatment. The pairwise comparisons were also done using the Dunnett post hoc test. The P values less than 0.05 were considered as statistically significant. The p-value less than 0.05, 0.01, 0.001, 0.0001 were marked as one to four stars (*).

## 3. Results

### 3.1. Structural characterization

**Fig 1:**
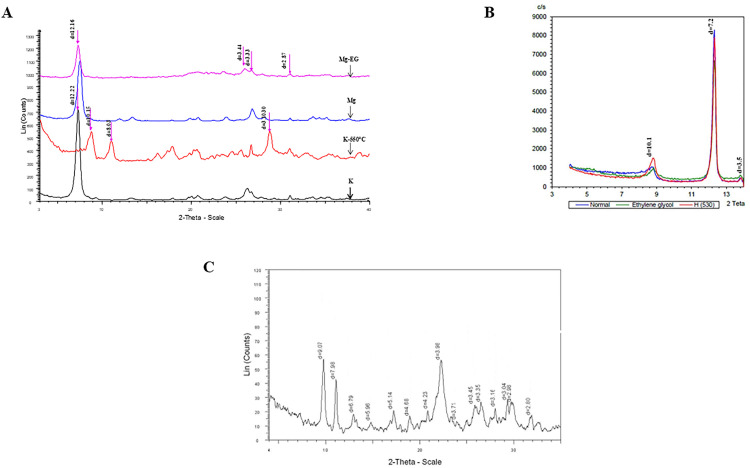
X.ray diffractogram of sepiolite (A), kaolinite (B), and zeolite (C). Mg: specimen saturated with MgCl_2_; K: specimen saturated with KCl; Mg-Eg: specimen saturated with MgCl_2_ and ethylene glycol; and K-550: specimen saturated with KCl after heating to 550 °C; d: d-spacing based on Angstrom (Å).

As illustrated in the XRD diffractograms showing the characteristic patterns of the investigated clays, intense peaks in 12.1 Å (related to sepiolite), 7.2 and 3.5 Å (related to kaolinite), and 4.2, 4.6, 5, 5.9, 7.9, 3, and 3.16, 3.3, 3.5, 3.71, 3.91, and 3.98 Å (related to clinoptilolite) confirm the presentation of this study’s clays (Fig 1A-C).

In detail, sepiolite was observed without any indication of substantial development of secondary phases (Fig 1A). Regarding other clay minerals, a small quantity of quartz and dolomite was detected in the sepiolite sample (Fig 1A). In Fig 1B, the peak at 10.2 shows that the kaolinite sample contains a small amount of illite (Fig 1B). Weak reflections at 4.2 and 3.3 show the presence of quartz and feldspar in the clinoptilolite sample, indicating only small quantities of these minerals (Fig 1C).

### 3.2. Chemical characterization

The results indicate that the CEC of kaolinite is 4.5 cmol (^+^)/kg and the CEC of sepiolite is 3.54 cmol (^+^)/kg. Regarding zeolite, the external CEC is 15.5 cmol (^+^)/kg and the internal CEC is 163.04 cmol (^+^)/kg.

The XRF measurements presented in Table 1 unequivocally demonstrate the lack of heavy metals in the studied clays.

**Table 1:** XRF analysis of sepiolite, zeolite, and kaolinite.

Clay minerals	SiO_2_%	Al_2_O_3_%	Fe_2_O_3_%	CaO%	MgO%	Na_2_O%	K_2_O%	TiO_2_%	MnO%	P_2_O_5_%
**Sepiolite**	55.4	0.6	2.04	2.56	38.9	0.01	0.09	0.06	0.02	0.01
**Zeolite**	55.7	13	4.7	1.5	3.9	2.9	0.3	–	–	–
**Kaolinite**	74.13	15.42	0.85	1.55	0.91	1.51	1.25	0.09	0.02	0.04

### 3.3. IC50 analysis

IC50 values related to sepiolite, zeolite, and kaolinite are shown in Table 2. Clay concentration for the treatment dose was determined considering IC50.

**Table 2:** IC50 analysis of the studied clays.

Clay minerals	IC50
**Zeolite**	**0.340**
**Sepiolite**	**0.097**
**Kaolinite**	**0.058**

### 3.4. Inhibitory effect of clays on cell viability

**Fig 2:**
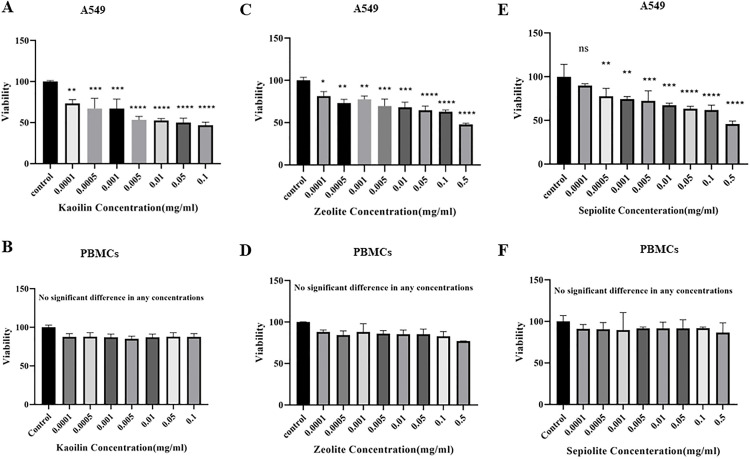
Viability rate of A549 cell line and PBMCs treated with different concentrations of kaolinite (A, B), zeolite (C, D), and sepiolite (E, F) compared to the untreated cells (*p ≤ 0.05, ** p ≤ 0.01, ***p ≤ 0.001).

Based on the results of the MTT test, Fig 2 shows how kaolinite, sepiolite, and zeolite affect the viability of the investigated cells. The results showed that zeolite, kaolinite, and sepiolite were generally safe for PBMCs while having a significant cytotoxic impact on A549 cells.

Fig 2 shows the viability rate of the A549 cell line and PBMCs treated with different concentrations of sepiolite, zeolite, and kaolinite compared to the negative control. Our findings showed that clay minerals reduced the viability of A549 cells while not affecting the viability of PBMCs. The IC50 values for zeolite, kaolinite, and sepiolite in the A549 cell line were calculated to be 0.34, 0.058, and 0.09 mg/mL, respectively. PBMC cells maintained a viability rate of 80–100% when exposed to varied concentrations of clay. The viability rate of A549 cells after being treated with different amounts of zeolite, kaolinite, and sepiolite was 47.7–81%, 46.7–73%, and 45–89%, respectively.

## 4. Discussion

The present study’s objective was to examine the potential anticancer effects of zeolite, sepiolite, and kaolinite on the viability of the A549 cell line using *in vitro* cytotoxicity assays compared to PBMCs. Our study findings showed that zeolite, kaolinite, and sepiolite were generally safe for human PBMCs, whereas they had a considerable cytotoxic impact on the cancerous A549 cell line. Kaolinite had the most toxicity among the three stated particles, with a significantly lower IC50 value. Furthermore, sepiolite was more toxic to the A549 cell line than zeolite. The internalization of kaolinite may play a crucial role in its cytotoxic effects. As previously mentioned, kaolinite can be found in particle sizes ranging from 0.3 to 100 micrometers [36]. The increased penetration of clay sheets into cells is possible. Furthermore, kaolinite led to an increase in the intracellular and extracellular production of superoxide radicals, thereby preventing metastasis [51].

The understanding of the complexity of cancer biology necessitates an in-depth comprehension of the specific functions of separate cell types inside the tumor and the surrounding tumor microenvironment (TME), which significantly influences the tumor’s unique characteristics [52]. The TME arises from the interplay between many cellular and non-cellular elements, such as cancer cells, stromal cells, and proteins [53]. The primary reason for the limited effectiveness of conventional anticancer treatments is the highly intricate nature of cancer biology [2].

Clays are showing enormous promise in cancer therapy as carriers for delivering anticancer drugs, as well as for their inherent ability to fight tumors, as demonstrated by some nano-clays [8]. Depending on the clay’s nature, it can have the highest level of porosity and a favorable correlation between physical and structural properties, resulting in the highest cell inhibition rates [9]. Each clay’s unique physical and chemical characteristics, such as morphology, shape, and CEC, influence its performance on different cells [54,55]. There are various morphologies of clays, including flat, film, and tubular, and their thickness ranges from 1 to 200 mm [56,57]. Particle size and structure can also improve cellular transportation and absorption [58–60]. Cellular physiological responses are significantly and directly influenced by their absorption [61]. Discrepancies in cell absorption and the way clays interact with the cell surface may account for variations in clay toxicity. Clays are valuable tools in the fight against cancer due to their ability to reduce acidity, enhance antioxidant capacity, and support apoptosis [62]. Clays may influence cancer growth by decreasing survival signals and inducing tumor suppressor genes in treated cells [15]. Additionally, clays regulate the levels of metabolic growth components [55].

Kaolinite’s internalization may play a critical role in its cytotoxic effects. As mentioned before, kaolinite could be found in particles ranging from 0.3 to 100 μm [36]. The increased penetration of clay sheets into the cells may be the cause of the cytotoxic effects of kaolinite. A study demonstrated that the “Kremnevit” kaolinite preparation exhibits anticancer properties, leading to a 24% decrease in tumor mass in cancerous mice as compared to non-cancerous mice. Furthermore, the treatment led to an elevated intracellular and extracellular production of superoxide radicals, thereby preventing metastasis [51]. A separate investigation involving sepiolite showed that after 30 minutes of exposure, sepiolite fibers were mainly concentrated on the cell’s outer surface. However, after 1 hour, the sepiolite was found on the surface and inside the cytoplasm. Ultimately, during 6 hours of interaction, the sepiolite was successfully identified within the cell. The transmission electron microscopy study also revealed the presentation of sepiolite fibers inside the endosomes, indicating that the fibers entered the cells by endocytosis when the cell membrane folds inward [63]. To date, no adverse effects have been observed with the oral and dermal consumption of clays. However, prolonged oral intake of clay may potentially lead to certain complications, the most significant of which include intestinal obstruction, anemia, and low potassium levels (hypokalemia) [64–66].

As previously stated, structures can impact the growth and division of cells. The distinct crystalline arrangement of different zeolites creates channels and cavities. Higher surface area and porosity increase the contact surface of the clay and consequently increase its absorption surface with the cell. Zeolites possess features that result in a small overall volume, allowing them to have a high capacity for adsorbing small molecules with low molecular weight [67]. Zeolite possesses distinct pore sizes and a well-defined structure of channels, which, depending on the size of the pores, can vary. This allows for the absorption of diverse small biomolecules [67]. The zeolite particles can adsorb significant quantities of macromolecules, including proteins [68]. Therefore, it can be speculated that the crystalline structure, pores, and well-defined channels in zeolite significantly inhibit the A549 cell line viability.

Clays have a distinctive characteristic known as CEC, which enables them to interact with cell surfaces and intracellular signaling pathways [69]. The CEC has two sources: A substitution may occur in either the tetrahedral or octahedral sheets. Replacing Al^3+^ with Mg^2+^ or substituting Si^4+^ with Al^3+^ results in a net negative charge. The mentioned section is regarded as stable since it exhibits minimal sensitivity to pH. Another source is the separation of aluminol groups located at the edges. The pH level influences the charges on these slightly acidic groups, leading to the pH-dependent CEC [70]. The interaction between the positively charged edges of the clay and the molecules in the cell membrane promotes the cells’ transport and uptake of the clay [59,71]. CEC facilitates interaction with cellular surfaces and intracellular signaling pathways [69]. The interaction of cationic edge charges in clay with cell membrane molecules enhances cellular uptake and absorption [59,71].

The present study findings showed that zeolite has an external CEC of 15.5 cmol (+)/kg and an internal CEC of 163.04 cmol (+)/kg, which is the highest among the investigated clays. Therefore, one could hypothesize that CEC might cause cell malfunctions, ultimately leading to cellular death.

Comparing the low concentrations of clay minerals in this study, the viability of kaolinite-treated cells was slightly lower than that of zeolite. However, in these low-concentration ranges, sepiolite did not have any detectable impact. Variations in the composition of clay, when introduced into a cell culture medium, may account for this variation. Based on the results of other research, even clinoptilolite, which is considered non-toxic and holds significant promise for various biomedical applications, has been shown to decrease cell viability and DNA synthesis while simultaneously increasing cell death. Clinoptilolite’s adsorptive and ion-exchange capabilities are responsible for the mentioned effects. This substance can remove certain components from the serum supplement in the cell culture medium, affecting calcium levels and calcium-dependent signaling pathways [69]. As a result, zeolite can be employed as a supplementary treatment in anticancer therapy [15]. Doxorubicin-intercalated kaolinite (DOX), with a diameter between 400 and 500 nm, revealed pH-sensitive behavior and antitumor activity towards ten tumor cell cultures [25]. An *in vitro* drug release kinetics study from the nanobiohybrid hydrogel with montmorillonite revealed a significant reduction in initial burst release and sustained release compared to the uncontaminated hydrogel. *In vitro* toxicity studies conducted on 293T cells with the MTT assay demonstrated that the nanobiohybrid hydrogel exhibits excellent biocompatibility. *In vivo* studies on anticancer efficacy conducted on mice carrying pancreatic cancer showed a significant reduction in tumor growth. Therefore, these results suggest that the nanobiohybrid hydrogel may represent a potential system for the controlled release of GEM in pancreatic cancer therapy, where the presence of montmorillonite is essential for efficient entrapment and for a controlled/prolonged release of the drug [72]. The MTT viability assay confirmed the reduced cytotoxicity to papillary thyroid cancer cells for methoxy-intercalated kaolinite [73].

According to our results, the inhibiting effect of all three clays was concentration-dependent. In a way, increasing concentrations led to a decrease in the cells’ viability. However, this effect was variable depending on the clay type as well. Kaolinite in equal and higher concentrations than 0.005 mg/mL demonstrated the most significant inhibitory activity against the A549 cell line. The concentration for sepiolite and zeolite was 0.05 mg/mL and 0.05 mg/mL, respectively. Given the study by Demircan et al. (2020), clinoptilolite was found to decrease cell proliferation dose-dependently in THP-1 cells after 24 hours of incubation under oxidative stress conditions. The use of clinoptilolite shows a concentration-dependent manner to inhibit the viability of diploid fibroblasts, cervical carcinoma, colon carcinomas, mammary carcinomas, and a mouse fibrosarcoma cell line [74]. In the research by Johari et al. (2021), the introduction of synthetic zeolite to HEK293 cells did not have a noticeable impact on cell viability at concentrations up to 62.5 µg/mL. However, when the exposure concentration was increased to 125 µg/mL, cell viability was significantly reduced to 36.69% compared to the control group (100%) [75]. Hoop et al. (2018) scrutinized the effects of varying concentrations of synthetic zeolite (ZIF-8 NPs) on six distinct human cell lines. Cell viability was around 80% when exposed to ZIF-8 concentrations of up to 30 µg/mL and decreased by 10% for 75 and 100 µg/mL [76]. “Comparisons with other clay minerals contextualize these findings. Montmorillonite (Mt) demonstrated concentration-dependent antiproliferative effects on MRC-5, HT-29, and HepG2 cell lines, influenced by protein levels and cell type [77]. Halloysite nanotubes (HNTs) showed low toxicity (≤20% viability reduction at 75 µg/mL) to HeLa and MCF-7 cells, with an IC50 ~ 300 µg/10^5 for A549 cells and nuclear localization without proliferation. Resveratrol-loaded HNTs enhanced MCF-7 apoptosis [78,79].

Another factor to consider is the aggregation of clay colloids, which occurs by increasing salt concentrations in the cell culture medium. Clays resist each other when dispersed in cell culture conditions due to their surface charge, preventing them from aggregating. The clays are effectively disseminated and carry a negative charge when they are in a diluted suspension. Nevertheless, when concentrations are elevated, the clays experience significant van der Waals pressures that cause them to stick together, as observed in the process of flocculation or aggregation [60]. Several studies [60,80–82] have indicated that under these circumstances, clay particles prefer to form micro-sized clusters or agglomerates and have a propensity to collect near cells. Accumulation can hinder cellular metabolism and cytoskeleton organization and obstruct membrane channels [83].

PBMCs are crucial in maintaining the body’s health under both normal and diseased conditions, playing key roles in immunity, infection control, cancer surveillance, and hematologic functions. Our findings regarding the minimal cytotoxic effects of clay minerals on PBMCs indicate their potential safety and immunocompatibility in therapeutic applications. By preserving the viability and function of these immune cells, clay minerals could support host immune defenses while targeting cancer cells selectively. Additionally, some clays may modulate immune responses by influencing cytokine production and macrophage activation, which may further enhance their anticancer efficacy and reduce adverse inflammatory effects. Thus, understanding these interactions is vital for advancing clay minerals as promising adjuncts or carriers in cancer therapy. The findings of the current study suggest that kaolinite, sepiolite, and zeolite clays are associated with favorable cytotoxic effects against the A549 cell line but not the PBMCs. In our study, PBMCs serve as a model for normal immune cells, comprising lymphocytes such as T cells, B cells, natural killer cells, and monocytes, which play critical roles in immune response. The observation of ‘no significant impact on PBMCs’ implies the absence of detectable cytotoxicity at the tested concentrations of zeolite, sepiolite, and kaolinite. Our finding was evidenced by maintained high viability rates (80–100%) of PBMCs in MTT assays. This selective cytotoxicity, where cancerous A549 cells are inhibited while normal immune cells remain viable, suggests a favorable safety profile of these clays for potential therapeutic applications. Furthermore, the results indicate that the performance of minerals varies in relation to different cell types. It appears that the adherent A549 cells provide greater opportunities for the penetration, accumulation, and ultimately the impact of materials, whereas the floating PBMC cells can remove substances from their surface due to their constant movement and displacement, thereby reducing efficacy. In fact, understanding the complexities of cancer biology necessitates a deep comprehension of the specific functions of distinct cell types within the tumor and the tumor microenvironment (TME), which significantly influence the unique characteristics of the tumor [52]. Cervini-Silva et al. (2017) investigated the role of the clay minerals sepiolite and bentonite on the proliferation behavior of cancer cell lines U251 and SKLU-1. Unlike fibrous clays, bentonite successfully enhanced the proliferation of SKLU-1 cells [54,55]. The effects of bentonite on various cell types were also examined by Nones et al. (2015), who stated that the effects of bentonite vary depending on concentration, exposure time, and the type of cells in contact with it [14]. In a recent study, Abduljauwad et al. used Na-MMT (Soidum Montmorillonite), Palygorskite, and hectorite with the aim of modulating the adhesion of tumor cells to the extracellular matrix. With their results, they have shown that Na-MMT is able to promote cell–cell adhesion, while palygorskite and hectorite favor cell adhesion to the extracellular matrix in Raji cells (lymphoma cell line). In addition, the combination of Na-MMT with Palygorskite (75:25) resulted in the greatest increase in cell–cell-extracellular matrix adhesions. Another wound healing test conducted with MCF-7 cells revealed that the nano-clays are able to control cell migration, resulting in a delay in gap closure, suggesting that the clay NPs tested could inhibit the migration of cancer cells with the prevention of metastasis formation [84]. In a study, it was shown that the “Kremnevit” kaolinite preparation shows an antitumor potential, resulting in a reduction in tumor mass (by 24%) in mice inoculated with cells of the LLC cell line (Lewis lung cancer) compared to non-cancerous animals [85]. The biocompatibility of the clayey mineral kaolinite was evaluated by Zhang et al. with respect to ten cancer cell lines, including pancreatic cancer, prostate cancer, lung cancer, stomach cancer, esophageal cancer, breast cancer, cancer of the cervix, and hepatocellular cancer. The cell viability rate was higher than 85% for most cell lines; that of esophageal cancer cells reached 99.8%, and that of the lung cancer cell line was only 61.3% [25,57]. Considering these results and the findings of the present study, the possibility of a specific effect on the abnormal cells is raised.

However, this effect also varied depending on the type and concentration of the clays. There is compelling evidence for the non-toxicity of clay particles at physiologically relevant doses on normal human or animal cells [69]. By way of example, the result of a study performed by Toledano-Magaña et al. (2015) demonstrated that the effect of clinoptilolite and sepiolite nanoclays on macrophage cultures depends on the cell’s origin. A small loss in viability (25%) occurred in the macrophages derived from human peripheral blood monocytes after 48 hours of exposure to nanoclays. The RAW 264.7 cell line experienced more decline (40%), while macrophages derived from mouse bone marrow monocytes were the most severely damaged (98%) [86]. Likewise, the antiproliferative effects of zeolite on human breast cancer MCF-7 cells have been investigated *in vitro* in the study by Subhapriya et al. (2018) [87]. Their findings showed that zeolite X inhibits the proliferation of MCF-7 cells and causes early apoptotic death through the activation of mitochondrial-dependent pathways, yet it does not promote cytotoxicity in MCF-12A non-cancerous cells. Interestingly, in the research by Cervini-Silva et al. (2016), which reported the impact of bentonite clays on cell growth behavior of U251 and SKLU-1 cancer cell lines, growth inhibition was observed in the U251 cells but not in the SKLU-1 cells, suggesting a highly particular interaction of bentonite with cell surfaces. The proliferation response of U251 cells was attributed to the regulation of metabolic growth factors by clay surfaces, resulting in the suppression of high-grade glioma formation. However, the increased growth response of SKLU-1 can be attributed to swelling, the aggregation of solutes, hydration, and modification through processes mediated by the clay surface [54].

In line with previous studies, we found that while all clays are able to affect the viability of cancer cells, this effect strongly depends on the type of clay or clay mineral. This result goes with another study by Cervini-Silva et al. (2017), reporting that all sepiolites either inhibited or increased the proliferation response of U251 or SKLU cells. Overall, the chemical composition was not sufficient to predict growth. Finally, whether differences in microporosity alter the behavior of cell proliferation is strongly dependent on the phyllosilicate being a clay mineral, such as sepiolite, or a clay such as bentonite [55]. The overall results of the effect of zeolite on pancreatic and cervical cancer cell lines indicated that an environment with a concentration of 50 mg/mL of zeolite exhibited the highest inhibition of cell proliferation [88]. In another study, natural zeolite (clinoptilolite) was investigated as a potential adjuvant therapy in cancer treatment, leading to improvements in overall health, longevity, and tumor size reduction in mice and dogs suffering from various types of tumors. The topical application of clinoptilolite in canine skin cancers effectively reduced tumor formation and growth. Furthermore, toxicological studies on mice demonstrated that this treatment had no negative effects [15]. Additional findings regarding the use of natural clinoptilolite as an anticancer agent have been corroborated by Zarković et al. (2003), who provided evidence for its efficacy [89]. It appears that halloysite, when applied at relatively high concentrations (greater than 0.1–0.2 mg/mL), is toxic to cancer cells [90]. The cytotoxic effect of kaolinite has been tested in A549 lung cancer cells, revealing an increase in DNA damage and a dose-dependent increase in the frequency of micronucleated A549 cells [91].

Given that the current study took place in an *in vitro* setting, we must acknowledge certain limitations. While the *in vitro* effect of the studied agents on human cell lines, which is very controllable and direct, will provide us with useful information about determining the optimal dosage and other characteristics of the clay, the complex interactions in living organisms are not fully replicable in cell culture models. Therefore, future studies are recommended to include *in vivo* animal models to assess the efficacy and systemic side effects of these clay minerals on all relevant tissues. Investigations involving additional normal cell lines, such as skin, lung, and mucosal cells, would improve the understanding of selective toxicity. Combination studies with established anticancer drugs may reveal synergistic effects or enhanced delivery capabilities. Moreover, while cytotoxicity was demonstrated by MTT assays, the underlying mechanisms responsible for the observed effects remain unknown. Future work should aim to elucidate whether apoptosis, necrosis, or reduced proliferation contribute to cytotoxicity and investigate associated pathways such as inflammatory responses and immune modulation. Positive control drugs such as doxorubicin were not employed in this study and are recommended for future research to validate assay sensitivity and contextualize potency. Ultimately, preclinical and clinical studies are essential to advance the translational potential of these promising agents. Notwithstandingly these *in vitro* findings provide a strong foundation for translational research. The next step involves validation in orthotopic lung cancer xenograft models to assess tumor regression, biodistribution, and immune modulation *in vivo*, building on successful nano-clay demonstrations in melanoma xenografts. Positive preclinical outcomes would support progression to Phase I clinical trials, initially testing safety and maximum tolerated doses of these Iranian-sourced clays in patients with advanced non-small cell lung cancer, potentially as adjuvants to standard chemotherapy.

Collectively, this work provides insights into the potential anticancer properties of kaolinite, sepiolite, and zeolite clays found in Iran and may also reveal new opportunities for developing therapeutic agents with safe, economical, and natural substances. We explicitly acknowledge that the current findings are restricted to the *in vitro* environment. The tested concentrations (which produced IC50 values of 0.340 mg/mL for zeolite, 0.058 mg/mL for kaolinite, and 0.097 mg/mL for sepiolite in A549 cells) do not directly reflect established, achievable concentrations in human plasma or tissue, which would define “physiologically relevant levels” for systemic exposure.

## 5. Conclusion

Our comparative analysis demonstrates that zeolite, sepiolite, and kaolinite exhibit cytotoxic effects against A549 lung adenocarcinoma cells while showing relatively less toxicity to PBMCs, which serve as representative immune cells rather than direct normal lung cell counterparts. These findings highlight the promise of these cost-effective and naturally abundant clay minerals as potential supplements or carriers in lung cancer therapy. Their unique physicochemical properties, including cation exchange capacity and favorable biocompatibility, support further investigation into their mechanisms of action and optimization for clinical translation. Future studies exploring combinations of these clays with established anticancer drugs are warranted to evaluate synergistic effects and enhance therapeutic efficacy. *In vivo* models and clinical evaluations will also be essential to fully realize their therapeutic potential and address safety considerations.

## Supporting information

S1 Data Supplementary Data. (XLSX)

S1 Fig Graphical abstract. (TIF)
