# Supplementary figures and images for: Comparative inhibitory effects of zeolite, sepiolite, and kaolinite on A549 lung cancer cells
Source: PLoS One. 2026 Feb 11;21(2):e0340270. doi: 10.1371/journal.pone.0340270 (PMC12893588; doi:10.1371/journal.pone.0340270)

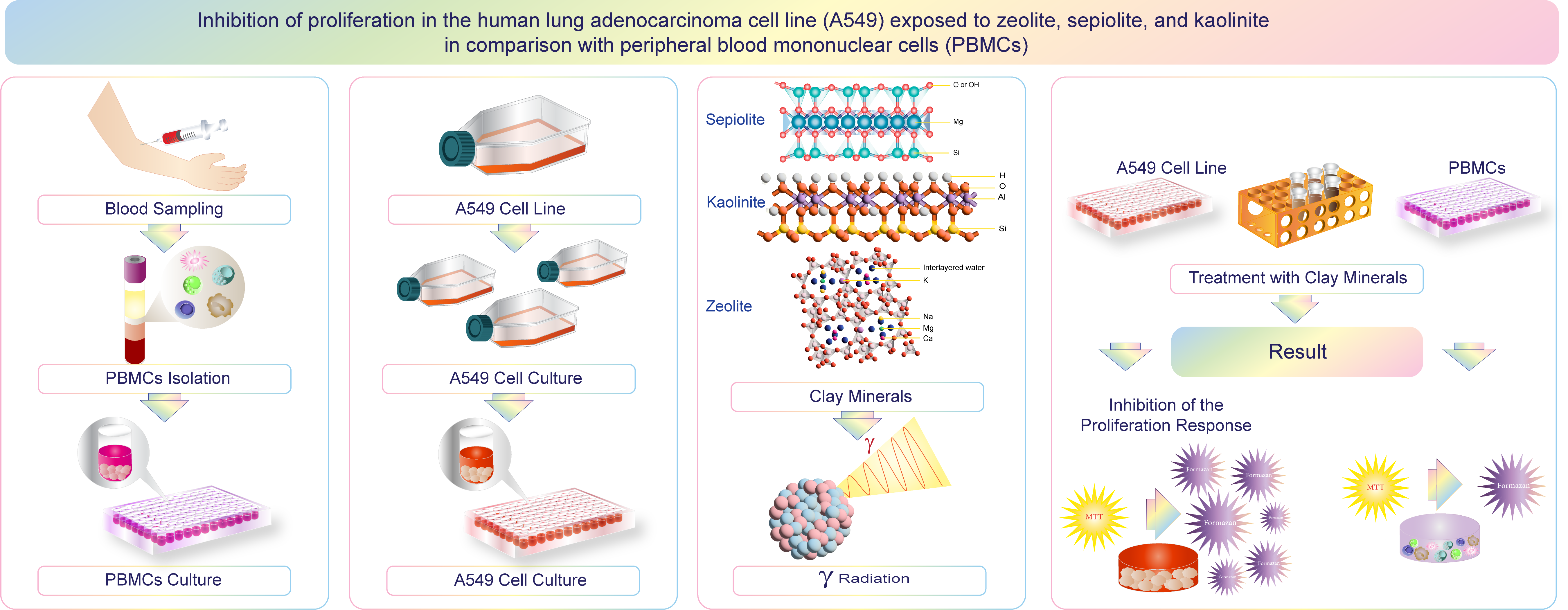

Supplement: S1 Fig — (TIF) [file pone.0340270.s002.tif]
